# Academic Integrity in Traditional Vs Online Undergraduate Medical Education Amidst COVID-19 Pandemic

**DOI:** 10.7759/cureus.13911

**Published:** 2021-03-15

**Authors:** Faiza Ikram, Muhammad Ali Rabbani

**Affiliations:** 1 Physiology, CMH Multan Institute of Medical Sciences, Multan, PAK; 2 Anatomy, CMH Multan Institute of Medical Sciences, Multan, PAK

**Keywords:** online learning, medical education, ethics, physiology, anatomy

## Abstract

Due to the coronavirus disease (COVID-19) pandemic, undergraduate medical education made a drastic paradigm shift towards online learning and assessment. This study was aimed at developing a statistical approach to empirically analyze the academic integrity of the online exams. Data were collected retrospectively from academic records of Physiology and Anatomy courses of a private medical college, in which students had attempted sequential on-campus and online modular exams. We developed a statistical model to predict on-campus and online exam scores of undergraduate medical students, from their previous academic records. Hypothesizing that the predictor model contains comparable explanatory power for both exams, we utilized the explanatory power variation (R^2^ statistics) of the improvised model to predict academic dishonesty behavior in traditional vs online exams. Reduced explanatory power (R^2 ^statistics) of the model for any mode of the exam, in which students scored considerably different from their previous academic record, was interpreted as indirect evidence of academic dishonesty. Our model explained a large proportion of variation (R^2^) in overall scores of on-campus and online Physiology and Anatomy modules. Whereas, the model could explain only a small proportion of variation in scores of online theory exams, with a moderate effect size (adjusted R^2^). Reduced explanatory power for both Physiology and Anatomy online theory exams implies the chance of academic dishonesty in this particular component of the online exam. Explanatory power variation of a predictor statistical model can be further explored and utilized to monitor academic integrity in future online exams.

## Introduction

Academic dishonesty has always been a challenge for educationists in various forms like plagiarism, fabrication, and cheating in exams or assignments. Multiple surveys have reported a high prevalence of self-reported academic dishonesty among healthcare students all across the globe [1, 2]. The estimated prevalence of cheating among medical students of the United States is 0% to 58% [3].

With the advancement in technology, traditional on-campus education is getting digitalized either to entirely online or hybrid modules. This educational revolution has further raised the concern of academic dishonesty regarding the new ways of e-cheating [4, 5]. Despite its utter importance, the available literature majorly highlights an abstract perception of ease and access to academic misconduct in online learning. Whereas, the factual data regarding the prevalence and quantification of academic misconduct in online learning is yet to be explored.

For the first time in 2008, Harmon and Lambrinos empirically analyzed cheating in proctored vs non-proctored online exams by using R-squared (R^2^) statistics of the academic performance predictor model [6]. They concluded that a relatively lower explanatory power of the prediction model for the non-proctored exam suggests that cheating has taken place. In 2009, Yates and Beaudrie compared the academic performance of students in parallel proctored and non-proctored online exams and concluded no higher incidence of cheating in non-proctored online exams [7]. In response to Englander’s [8] commentary on potential biases in Yates and Beaudrie's study [7], Beck [9] in 2014 empirically analyzed dishonesty behavior in online examination while using R^2^ statistics of academic performance predictor model. They didn’t find any evidence of higher academic dishonesty in unmonitored online exams.

Pakistan is a lower-middle socioeconomic country [10], with an exclusive traditional on-campus education system, specifically in the field of undergraduate medical education. In addition to the scarcity of resources and IT infrastructure, lack of training and acceptance among students and faculty, and fear of compromised academic integrity are also the barriers towards digitalization of medical education [11, 12]. Despite these challenges, during the recent era of the COVID-19 pandemic, undergraduate medical education was shifted entirely to the online learning program in Pakistan. Being supported by many, the online medical education system still had to face lots of criticism about the self-presumed compromised academic integrity in the online learning and examination system [13]. Although well debated, there is a huge research gap in empirically analyzing the extent of academic dishonesty behavior in online vs traditional examination of medical students.

Carrying on the Harmon and Lambrinos concept we intended to design an academic performance predictor model for undergraduate medical students while using the previous academic performance indicators [6]. Secondly, we intended to utilize the improvised academic performance predictor model to empirically analyze the academic dishonesty behavior in online vs traditional exams. Once established, this statistical model can be utilized by policymakers in future online medical education to monitor and minimize academic dishonesty. We hypothesized that our predictor model contains approximately equal explanatory power for on-campus and online exams. And if cheating takes place, the explanatory power (R^2^) of the predictor model will decrease for that exam. To our knowledge, this study is unique in empirically analyzing academic dishonesty in online vs on-campus exams among undergraduate medical students.

## Materials and methods

This comparative observational study was carried out retrospectively on academic record data of Second (2nd) year undergraduate students of a private medical college. In our institute, Anatomy and Physiology courses are conducted in three modules, followed by a summative modular exam at the end of each module. In 2020, the first module of 2nd year Bachelor of Medicine, Bachelor of Surgery (MBBS) was carried out conventionally, including on-campus lectures, tutorials, small group discussions, practical classes, and internal assessment class tests/assignments. The first (1st) modular exam was also carried out as a traditional on-campus exam, consisting of theory (multiple choice and short essay questions), practical, and viva exam. Whereas, the second module of 2nd year MBBS was shifted towards an exclusively online paradigm due to the COVID-19 pandemic. Using the personal accounts on the ‘Google classroom’ platform, students were provided learning materials on a regulated schedule via recorded video lectures, PowerPoint presentations with audio narration, and relevant hand-outs. Alongside the flexible discussion board on google classroom, students had daily timed interaction with their instructors via personal meeting accounts on ‘Zoom’ in form of short groups. Internal assessment assignments of the second module were also carried out online. By the end of the module, the summative modular theory exam was conducted digitally using timed google forms including multiple choice and short essay questions. Students had one-time access to the online exam. Students had to show their identity before starting the exam and their continuous video monitoring on live ‘Zoom’ session was ensured via trained instructors. Although, no special software was used to monitor their screen activity. The module viva exam was also conducted digitally via ‘Zoom (version 5.2)’. For both the courses, the instructors and examiners were the same as the previous module.

Data was obtained for 97 (39 male and 58 female) students, while four students of 2nd year MBBS were excluded from the study who had not attempted either of the modular exams. Percentage scores were obtained for modular exams, internal assessment, and previous year’s (1st year MBBS) final examination along with students’ gender information. The study was planned on retrospective exam data to avoid potential bias in exam performance and scoring.

Designing academic performance predictor model

Using modular exam scores as a performance indicator, the statistical model was improvised to predict the academic performance of students in exams. Following the literature review [14-16] the academic performance model was based on three predictors: the Previous year’s final examination score (comparable to cumulative GPA), internal assessment of the respective module, and gender. Being a less versatile study group from a small population of a single institute, demographic variables like age, ethnicity, socioeconomic and residential status were not included in the model. Also, non-verifiable and subjective indicators like motivational, behavioral, and situational factors were not included in the model. The equation used for predicting academic performance in online and on-campus exams of students was:

Exam (i) = b0 + (b1 x Gender) + (b2 x 1st Year Exam) + (b3 x Internal Assessment)

Statistical analysis

Statistical analysis was carried out using Statistical Package for the Social Sciences (SPSS) version 20 (IBM Corp., Armonk, NY). Multiple regression analysis was carried out to predict scores of on-campus and online modular exams for Physiology and Anatomy courses separately, using the academic performance indicator model described above. As the theory exam is specifically vulnerable to cheating as compared to the viva exam, a similar regression analysis was carried out to predict the only theory exam scores for on-campus and online modular exams. The linearity and homoscedasticity of data were assessed by inspecting plots of studentized residuals against predicted values. The independence and multicollinearity of residuals were assessed by values of Durbin-Watson statistic (close to 2) and tolerance values (>0.1), respectively. The normality of data was assessed by Q-Q plots visual inspection.

R-squared statistics (R^2^) were computed to compare the explained variance in online vs on-campus modular exams, for Physiology and Anatomy courses separately. We hypothesized that the predictor model should equally explain variation in online and on-campus exam scores. Whereas, lower R^2^ statistics for one mode of the exam would mean that students have scored considerably different from what was predicted from their previous academic record. Thus, the lower explanatory power of the model for an exam was interpreted as cheating has taken place.

## Results

Descriptive statistics of participants’ exam scores in the Physiology and Anatomy course are given in Table 1, for online and on-campus modular exams.

**Table 1:** Percentage Mean Scores of 2nd year MBBS Students in Online and On-campus Modular Exams ^†^Past Exam; Previous year final exam, ^‡^IA; Internal Assessment

Exam	Component	Physiology Scores (%)		Anatomy Scores (%)	
		Mean	Standard Error	Mean	Standard Error
	Past Exam^†^	67.47	0.71	71.48	0.78
On-Campus Module	IA^‡^	47.74	2.1	51.59	2
	Overall Exam	54.85	1.41	64.2	1.51
	Theory Exam	48.86	1.42	66.2	1.57
Online Module	IA^‡^	68.73	1.84	81.19	1.32
	Overall Exam	64.53	1.59	63.3	1.43
	Theory Exam	69.6	1.2	58.5	1.27

Regression analysis was carried out after satisfying the assumptions. The multiple regression model statistically significantly predicted overall and theory exam scores of on-campus and online Physiology modules. The coefficient of determination (R^2^) showed that our model explained 80% and 78% variation (R^2^) in overall and theory exam scores of the on-campus module, with a large effect size (ΔR^2^) of 79% and 78% respectively, according to Cohen [17]. The predictor model also explained a 50% variation in the overall exam of the online Physiology module, with a large effect size of 49% [17]. However, the model could only explain a 20% variation in the theory exam of the online Physiology module, with a medium effect size of 18% [17]. Previous year exam scores and internal assessment added a statistically significant contribution to the prediction of on-campus exam scores. Whereas, all three predictors contributed significantly to predicting online Physiology module scores. Regression coefficients and confidence intervals for individual predictors are given in Table 2.

**Table 2:** Previous Academic Record as Predictor of On-campus and Online Physiology Exam Scores R^2^: Coefficient of determination, ΔR^2^: Adjusted R^2^, p: significant at <0.05, B: Unstandardized Regression Co-efficient, CI: Confidence Interval, β: Standardized Coefficient, *Gender: Coded as “0” for male and “1” for female, †Past Exam: Previous year Final Exam, ‡IA: Internal Assessment

Physiology	On-Campus Module Exam				Online Module Exam			
	R	R^2^(ΔR^2^)	F	p	R	R^2^(ΔR^2^)	F	p
Model	0.89	0.80(0.79)	122.32	0.000	0.71	0.5(0.49)	31.15	0.000
	B	95% CI (B)	β	P	B	95% CI (B)	β	P
Constant	-5.44	-20.61±9.73		0.48	-3.09	-26.07±19.89		0.79
*Gender	1.35	-1.35±4.04	0.05	0.32	-1.99	-7.31±3.33	-0.06	0.46
^†^Past Exam	0.54	0.29±0.78	0.27	0.000	0.48	0.14±.82	0.21	0.01
^‡^IA	0.46	0.37±0.54	0.68	0.000	0.56	0.41±.71	0.65	0.000
Physiology	On-Campus Module Theory Exam				Online Module Theory Exam			
	R	R^2^(ΔR^2^)	F	p	R	R^2^(ΔR^2^)	F	p
Model	0.89	0.78(0.78)	111.57	0.000	0.45	0.2(0.18)	7.89	0.000
	B	95% CI (B)	β	P	B	95% CI (B)	β	P
Constant	-15.06	-30.84±0.71		0.06	45.47	23.57±67.39		0.000
*Gender	3.71	0.9±6.51	0.13	0.01	-6.1	-11.18±-1.03	-0.25	0.02
^†^Past Exam	0.55	0.3±0.81	0.28	0.000	0.2	-0.13±0.52	0.12	0.23
^‡^IA	0.44	0.35±0.52	0.65	0.000	0.3	0.16±0.44	0.46	0.000

The multiple regression model statistically significantly predicted overall exam scores of on-campus and online Anatomy modules. Regression analysis, however, failed to predict theory exam scores of the online Anatomy module significantly. The coefficient of determination (R^2^) showed that the model explained 89% and 80% variation in overall and theory on-campus modular exams respectively, with large effect size (ΔR^2^) according to Cohen [17]. Whereas, the predictor model could explain 39% and 8% variation in overall and theory exam of the online Anatomy module, with large (37%) and moderate (5%) effect size respectively [17]. Previous year exam scores and internal assessment added a statistically significant contribution to the prediction of overall module scores, but insignificantly towards online theory exam. Regression coefficients and confidence intervals for individual predictors are given in Table 3.

**Table 3:** Previous Academic Record as Predictor of On-campus and Online Anatomy Exam Scores R^2^: Coefficient of determination, ΔR^2^: Adjusted R^2^, p: significant at <0.05, B: Unstandardized Regression Co-efficient, CI: Confidence Interval, β: Standardized Coefficient, *Gender: Coded as “0” for male and “1” for female, †Past Exam: Previous year Final Exam, ‡IA: Internal Assessment

Anatomy	On-Campus Module Exam				Online Module Exam			
	R	R^2^(ΔR^2^)	F	p	R	R^2^(ΔR^2^)	F	p
Model	0.95	0.89(0.89)	256.83	0.000	0.62	0.39(0.37)	19.81	0.000
	B	95% CI (B)	β	P	B	95% CI (B)	β	P
Constant	-5.62	-16.99±5.75		0.33	-26.22	-49.69±-2.75		0.03
*Gender	0.19	-1.88±2.27	0.006	0.85	5.31	0.38±10.24	0.19	0.04
^†^Past Exam	0.59	0.4±0.77	0.3	0.000	0.88	0.57±1.18	0.48	0.00
^‡^IA	0.54	0.47±0.61	0.71	0.000	0.23	0.03±0.42	0.21	0.02
Anatomy	On-Campus Module Theory Exam				Online Module Theory Exam			
	R	R^2^(ΔR2)	F	p	R	R^2^(ΔR2)	F	P
Model	0.9	0.8(0.8)	126.31	0.000	0.27	0.08(0.05)	2.51	0.06
	B	95% CI (B)	β	P	B	95% CI (B)	β	P
Constant	-2.08	-18.12±13.96		0.8	48.79	23.18±74.41		0.000
*Gender	-0.7	-3.63±2.22	-0.02	0.63	-5.77	-11.15±-0.4	-0.23	0.04
^†^Past Exam	0.59	0.33±0.84	0.29	0.000	0.27	-0.06±0.61	0.17	0.11
^‡^IA	0.53	0.43±0.63	0.68	0.000	-0.01	-0.21±0.2	-0.01	0.96

## Discussion

Since the digitalization of the education system, online course delivery, and examination have been maligned for compromised academic integrity [18, 19]. Unfortunately, most of this debate is speculative rather than being empirical [20]. This study was a step towards filling this empirical research gap. Our study showed that preceding academic performance indicators like previous exam scores and internal assessment scores can significantly predict academic performance in on-campus or online exams. Our study proposed that explanatory power variation of predictor models can be utilized to monitor and predict academic dishonesty behavior in the online vs face-to-face exams, among undergraduate medical students.

Our model explained a large proportion of variations, with a large effect size, in overall exam scores of on-campus and online Physiology modules. Our model also significantly explained variation in online theory exam with large effect size, however, it could explain considerably less variance in online theory exam with a moderate effect size. Reduced model explanatory power and effect size for the online theory exam suggests that students have scored significantly different from their predicted values, hence cheating has taken place. Adequate prediction of overall exam score of online Physiology module with large effect size indicates that components of exam other than theory (e.g., viva) may help maintain the academic integrity. To further validate our results, the same model was utilized to predict online and on-campus module results of a parallel Anatomy course. Again, the model showed a decreased model effect size only for the online theory exam. This indicates a higher incidence of cheating, particularly in the theory component of the online exam. These results imply that online examinations can be properly designed to decrease academic misconduct.

Previously, studies using the same statistical approach to predict academic dishonesty in online exams have shown inconsistent results. In 2008, an economics course study concluded a higher incidence of cheating in non-proctored vs proctored online exams [6]. Whereas a similar study in 2014 concluded that unmonitored online exams had no higher incidence of cheating as compared to the monitored online exams [9]. Our study supports the findings of Harmon and Lambrinos [6]. However, our study is different in utilizing the preceding academic performance markers to predict exam scores. Also, we have compared on-campus vs traditional exams rather than monitored and unmonitored online exams. Also, our study is one step ahead in empirically comparing the online and on-campus exams while considering components of exams other than theory also. We couldn’t find a comparable study empirically comparing traditional and online exam outcomes of undergraduate medical students.

In our study, previous year exam scores, internal assessment, and gender variably contributed to the prediction of exam scores. These results are supported by a multitude of previous researches [14-16]. Our proposed model has predicted a huge proportion of variation in exam scores with a large effect size. This model can be used in future studies to monitor the integrity of online exams among undergraduate medical students. The given model can be further refined as per previous literature [6, 9] to include human capital variables like age, ethnicity, earned credit hours, residential and socioeconomic status, for a larger sample size.

This study was carried out retrospectively on two consecutive modules of undergraduate Physiology and Anatomy courses, conducted on-campus and online sequentially. Instructors, examiners, and participants of both modules were the same, thus reducing the selection, performance, and observer bias. Whereas the course and mode of conduction of module and exam were different, which could have led to the potential bias. The on-campus exam was precisely monitored to reduce cheating, whereas the online exam was monitored only via live video sessions with verbal warnings. Because of a lack of funds and resources, no specialized software could have been utilized for proctoring the online exam. Despite repeated reminders regarding strict action against academic misconduct, online exam in given resources might have been circumstantially more favorable for cheating as compared to the traditional exams.

The strength of our study is empirically analyzing academic dishonesty behavior among undergraduate medical students while comparing across different components of online and traditional exams. Another strength of our study is the inclusion of a learning module that was both conducted and accessed online in limited resources. So, the proposed model can be replicated in other setups with limited resources. However, our study sample is from a small population of a single institute. So, more studies should be conducted to support and generalize our findings.

The observed increased incidence of cheating in online theory exams cannot be purely contributed to online education. Our study supports previous studies emphasizing that designing of online course and exam, conduction, and monitoring should be modified accordingly to preserve academic integrity [21]. As academic dishonesty behavior is inevitable in any mode of examination, the statistical model proposed in our study can be utilized in future researches to predict and monitor cheating in online exams of undergraduate medical students. Hence, the stakeholders of online medical education like policymakers can utilize this sophisticated statistical approach to minimize academic misconduct in online medical education.

## Conclusions

A preceding academic record like internal assessment and previous exam results can significantly predict scores of undergraduate medical students in traditional on-campus exams. This explanatory power variation of a predictor statistical model can be further explored and utilized to monitor academic integrity in future online exams.
